# Notalgia Paresthetica: A Novel Approach to Treatment with Cryolipolysis

**DOI:** 10.7759/cureus.1719

**Published:** 2017-09-28

**Authors:** Philip R Cohen

**Affiliations:** 1 Department of Dermatology, University of California, San Diego

**Keywords:** cryolipolysis, itch, nerve, notalgia, pain, paresthetica, pruritus

## Abstract

Notalgia paresthetica, a neurosensory syndrome that typically occurs on the upper back, has multiple clinical symptoms with variable degrees of expression in each individual afflicted with the condition. The involved site is usually hyperpigmented and is associated with burning, coldness, hypoesthesia, increased pain, pruritus and/or tingling. In the affected area, the number of nerve fibers may be increased and the cutaneous sensory nerves are altered secondary to localized impingement, central injury, or both. Although multiple therapeutic approaches for notalgia paresthetica have been described, none specifically address the essential pathogenesis of the condition—the altered cutaneous nerves. Cryolipolysis is a well-tolerated nonsurgical technique to reduce the subcutaneous fat layer. Selective apoptosis of adipocytes occurs since the lipid-rich fat cells are more susceptible to cold injury than the surrounding water-rich cells. Not only a marked decrease in pain sensitivity but also a sustained reduction in the density of myelinated and unmyelinated cutaneous nerves has been observed in cryolipolysis-treated skin. Therefore, cryolipolysis is a logical approach to the treatment of notalgia paresthetica. One or more cryolipolysis treatments may be necessary for complete or partial resolution of the individual’s notalgia paresthetica-related cutaneous symptoms. In conclusion, evaluation of cryolipolysis as a noninvasive treatment of patients with notalgia paresthetica is warranted.

## Introduction and background

Notalgia paresthetica is a neurosensory condition that typically occurs on the upper back. The affected area is often hyperpigmented and associated symptoms—restricted to the involved site—include not only pruritus, but also burning, coldness, hypoesthesia, increased pain, and tingling. There is an alteration of the cutaneous sensory nerves resulting from either localized impingement, central damage and irritation, or both; in addition, an increased number of nerve fibers may be present [1-4].

Cryolipolysis is a well-tolerated nonsurgical procedure to reduce the subcutaneous fat layer. Selective apoptosis of adipocytes occurs since the lipid-rich fat cells are more susceptible to cold injury than the surrounding water-rich cells. A sustained decrease of myelinated and nonmyelinated sensory cutaneous nerves, along with decreased vibratory and thermal pain, has been demonstrated in skin following cryolipolysis [5-7].

Topical, intralesional, systemic, peri-neural, and non-pharmacologic approaches to the treatment of notalgia paresthetica have been performed with variable—limited duration or long-term—efficacy [8]. However, none of the therapeutic interventions utilized in notalgia paresthetica directly addresses the essential pathogenesis of the condition—the altered nerve. Cryolipolysis—which specifically results in decreasing cutaneous sensory nerves, may be a novel modality for the successful management of notalgia paresthetica.

## Review

Notalgia paresthetica

Background

Notalgia paresthetica, a sensory neuropathic syndrome, is characterized by a single or multiple affected areas on the back that exhibits one or more of the following symptoms: pain, burning, a cold sensation, hyperesthesia, hypoesthesia, numbness, paresthesias, and tingling. Pruritus is also typically present. Hyperpigmented macules, overlying the symptomatic site, may also be observed [1-2].

The condition was initially described in St. Petersburg Russia by a neurologist, Dr. Astwazaturo, in 1934 [1, 3]. The term notalgia is derived from the Greek words 'notos,' meaning back, and 'algos,' meaning pain. The nomenclature emphasizes the combination of symptoms that are manifested: back pain and paresthesia [3-4].

Epidemiology

Notalgia paresthetica is a common condition that is encountered worldwide [3, 9]. There is no racial predilection [3, 10]. It can occur in any adult age group; however, it most frequently occurs in women between the ages of 54-62 years old [10]. Notalgia paresthesia has also been observed in patients with multiple endocrine neoplasia type 2a (MEN 2A); in these individuals, the condition occurs in younger patients—even children [11].

Clinical Presentation

The sensory symptoms typically present within a single area, often located on the upper back; the upper and lower borders are between thoracic level two (T2) and thoracic level six (T6), and the lateral borders are between the vertebra and the scapula. A prior history of herpes zoster in the affected area is absent. Usually, the condition is unilateral; rarely it is bilateral. A hyperpigmented patch, with or without accompanying hyperkeratosis or lichenification, may be present at the site of the symptoms [1-4, 9, 10, 12-14].

Pathology

A biopsy of the hyperpigmented patch of notalgia paresthetica shows mild hyperkeratosis of the epidermis; the papillary dermis shows a mild lymphocytic inflammatory infiltrate and melanophages. These changes are consistent with those observed in post-inflammatory hyperpigmentation. In addition, necrotic keratinocytes or amyloid deposits in the papillary dermis (consistent with macular amyloidosis), or both, may be present. If necessary, a biopsy may aid to differentiate notalgia paresthetica from some of the other conditions in the clinical differential diagnosis, such as contact dermatitis, fixed drug eruption, macular amyloidosis, neurodermatitis, parapsoriasis, post-inflammatory hyperpigmentation, and tinea versicolor [2, 4, 8, 13].

Pathogenesis

Notalgia paresthetica is a sensory neuropathy that results from an alteration of the cutaneous sensory nerves of the upper back. The nerve alteration may be secondary to either localized impingement (possibly from the adjacent muscles) or central damage and irritation (related to pathologic processes of the spine) or both [1, 4]. In addition, albeit less well supported, there is evidence that the localized area of symptoms has an increased number of nerve fibers [15-16].

The dorsal segments of the thoracic nerves of T2 to T6 are susceptible to trauma and entrapment by the spinal muscles since they pass through the muscles at a right angle after exiting from the spinal cord, resulting in notalgia paresthetica. Nerve injury to nearby nerves that affect muscles in the area of symptoms can also result in the condition; for example, dysfunction of the serratus anterior muscle following injury to the long thoracic nerve caused notalgia paresthetica. A similar mechanism of pathogenesis could be the sequelae of injury—such as damage to the trapezius muscle that results in instability of the scapula and subsequent adverse effects on the cutaneous nerves [17-18].

Spinal pathology can cause direct damage to the nerves and result in notalgia paresthetica. Several structural changes have been observed in patients with this condition: degenerative changes, disc herniation, kyphosis, osteoarthritis, scoliosis, spinal stenosis, and vertebral arthrosis and hyperostosis [9, 14]. In addition, referred pain to the upper thoracic and infrascapular areas secondary to cervical spine disease may also be a potential etiology of notalgia paresthetica [19].

The pathogenesis of notalgia paresthetica in patients with MEN 2A is hypothesized to be different. The patients are younger and do not have the spinal pathology that has been observed in older individuals with the condition. Signaling of neural crest cells by RET/glial cell line-derived neurotrophic factor is postulated not only to cause other manifestations of MEN 2A, but also to promote the development of notalgia paresthetica in MEN 2A patients [11].

The etiology of the hyperpigmented patch of notalgia paresthetica remains to be definitively established. Some investigators consider the friction from chronic rubbing to result in post-inflammatory changes with concurrent basal keratinocyte apoptosis and secondary amyloid deposition [20]. However, other researchers theorize that the presence of amyloid is part of the primary disease process and causes the pigmentary changes [4]

Treatment

Several modalities have been utilized for the management of patients with notalgia paresthetica. They include topical, oral, and interventional therapies; the latter include intralesional, non-pharmacologic, and peri-neural therapeutic treatment options (Table 1) [4, 10-11, 13, 18-19, 21-37]. However, a treatment such as cryolipolysis, that could actually modify the cutaneous nerves at the site of the symptoms, might be effective in temporarily alleviating or permanently resolving the condition.

**Table 1:** Notalgia paresthetica: therapeutic treatment options

Treatment	References
Intralesional	
Botulinum toxin	[21,22]
Corticosteroids	[13]
Non-pharmacologic	
Acupuncture	[11]
Exercise	[23]
Latissimus dorsi and rhomboid muscle strengthening	[23]
Pectoral muscle stretching	[23]
Massage	[19]
Multimodal physiotherapy (infrared, radar, short waves and ultrasound)	[10]
Narrow band ultraviolet-B	[24]
Osteopathic manipulative treatment	[25]
Inhibition and soft tissue techniques	[25]
Rib raising	[25]
Scapulothoracic fascial release	[25]
Suboccipital decompression	[25]
Surgery (decompression of cutaneous nerve or discectomy)	[26]
Transcutaneous electrical muscle stimulation (EMS)	[18]
Transcutaneous electrical nerve stimulation (TENS)	[27]
Oral	
Antidepressants	[4,28]
Selective serotonin norepinephrine reuptake inhibitors	[4,28]
Tricyclic antidepressants	[4,28]
Antihistamines (sedative (histamine one) H1 and/or non-sedative H1)	[4]
Gabapentin	[29,30]
Muscle relaxants	[19]
Nonsteroidal anti-inflammatory drugs	[4]
Oxcarbazepione	[31,32]
Pregablin	[29]
Peri-neural	
Paravertebral nerve block (bupivacaine and methylprednisolone)	[33]
Topical	
Anesthetics (lidocaine 2.5% plus prilocaine 2.5% cream)	[34]
Capsaicin (0.025% and 0.075%) cream	[35,36]
Corticosteroids	[10]
Naltrexone 1%	[31]
Tacrolimus	[37]

Cryolipolysis

Background

Cosmetic procedures focused on body contouring continue to increase in popularity. Side effects from invasive surgical procedures (such as liposuction) for fat removal have prompted the development of several noninvasive body contouring devices. Cryolipolysis is a noninvasive procedure for subcutaneous fat layer reduction [5-6].

Cryolipolysis initially received clearance by the Food and Drug Administration (FDA) to treat the flank area (love handles) in 2010. Subsequently, FDA clearance has followed to include subcutaneous fat treatment for the abdomen, the thighs, and the submental area in 2012, 2014, and 2015, respectively. More recently, in 2016, clearance was received for the arms, back, bra area, and the area beneath the buttocks [5, 10].

Procedure

The cryolipolysis device consists of an applicator that is attached to a control unit. A coupling gel is applied to the skin site and the cup-shaped applicator is applied. A moderate vacuum within the applicator draws the tissue between two cooling panels. The cooling process is accelerated by the vacuum-associated constriction of blood vessels. Cooling of the skin (measured by the selected heat extraction) is controlled by sensors that monitor the heat flux out of the skin and is modulated by a thermoelectric cooling element [6].

Cryolipolysis is operator independent once the applicator has been affixed to the site of therapy. The cycle duration of treatment is up to one hour. The cooling of the skin incorporates a predetermined energy extraction rate that is expressed as a numeric value denoted by the cooling intensity factor (CIF) [6].

Immediate, and transient, post-cryolipolysis adverse events at the treatment site typically include edema and erythema. Several patients also experience pain in the area treated; yet, by post-treatment day seven, all patients are usually free of these symptoms. However, a common side effect--which typically resolves one to two months after cryolipolysis--is prolonged hypoesthesia in the treated area [7].

Observations

Subcutaneous fat is sensitive to cold-induced injury. Indeed, compared with other water-rich cells, adipocytes have a higher sensitivity to cold. Therefore, cryolipolysis results in a selective reduction of subcutaneous fat layer thickness without any physical damage to the surrounding tissue [5-6].

Cryolipolysis does not affect serum lipid levels or liver function tests; indeed, treatment of multiple sites on the same day is safe [38]. Also, recent adaptations to the cryolipolysis applicator have not only maintained the safety and efficacy of the procedure, but also decreased the treatment duration and allowed the procedure to be more comfortable due to lower vacuum-skin tension [39]. In addition, animal studies have now shown that cryolipolysis-associated fat reduction efficacy was greater when combined with tumescent-solution injection [40].

Cryolipolysis and Sensory Nerve Function

Coleman, et al., in 2009, published their study regarding the clinical efficacy of noninvasive cryolipolysis and its effects on peripheral nerves. A cooling device was used to treat areas on one of two contralateral flanks in 10 subjects. Sensory function was assessed by neurologic evaluation in nine of the subjects (who had been treated for 60 minutes) and by skin biopsy for nerve staining in one subject  (who had been treated for 45 minutes) [41].

In six of the nine subjects, there was a transient reduction in sensation. Specifically, reduction in sensitivity to pain or pinprick was noted in all six subjects one week after treatment. The altered sensitivity lasted between one to six weeks; however, by two months after treatment, all reduction in pain sensitivity had resolved [41].

Serial skin biopsies (from treated and control sites) were evaluated before treatment and three months after treatment in one subject. All biopsies showed an equal and normal number of epidermal nerve fibers and nerve plexi, suggesting that cryolipolysis did not cause any long-term change to the structure or functionality of either the epidermal nerve fibers or nerve plexi in the dermis [41]. In addition, neither inflammation nor necrosis of nerves was observed; however, the study did not address the neurosensory pathways and mechanisms involved [7]. 

Garibyan, et al., in 2015, conducted a larger study to assess alterations of cutaneous nerve function by noninvasive cryolipolysis. They evaluated pain, itch, and cutaneous nerve fiber density over a 56-day follow-up period after a single cryolipolysis treatment. Six women and five men were treated with cryolipolysis for one hour on their flank [7].

A marked decrease in pain sensitivity--not only mechanical but also thermal—was produced by cryolipolysis. Within two to seven days following treatment, the hyposensitivity began; it persisted for at least 35 days after cryolipolysis. In contrast, there was no significant change in the mean or peak itch intensity after cryolipolysis [7].

Serial skin biopsies (at baseline, 48-72 hours, 21 days, and 56 days post-treatment) were performed in six subjects. The density of dermal myelinated nerve fibers and unmyelinated epidermal nerve fibers were analyzed. In contrast to the earlier study by Coleman, et al. [41], the investigators found that there was a prolonged reduction in the density of both myelinated and unmyelinated cutaneous nerves following cryolipolysis; the former group of nerves in the dermis was most greatly affected [7].

The postulated pathogenesis of cutaneous sensory nerve alteration varied depending on whether the affected nerves were myelinated or unmyelinated. Cryolipolysis preferentially injures myelinated dermal nerves since they contain a lipid-rich myelin sheath; subsequently, the peripheral nerve function decreases. The cryolipolysis-induced changes of unmyelinated nerves are hypothesized to possibly be multifactorial: cold-induced lipid crystallization, ischemia-reperfusion injury, metabolic stress, and/or stress signaling pathways [7].

## Conclusions

Notalgia paresthesia is a common sensory neuropathic syndrome having multiple clinical symptoms with variable degrees of expression in each individual with the condition. Multiple treatment modalities have been reported; however, none of the therapeutic approaches focus directly toward the principle etiology of the condition: the affected cutaneous nerves. Cryolipolysis is a noninvasive technique utilized for the reduction of subcutaneous fat. An unexpected observation following cryolipolysis was a marked decrease in pain sensitivity of the treated skin. Cryolipolysis of notalgia paresthetica sites on the back of affected individuals is a logical approach to the management of this prevalent condition. In summary, evaluation of a noninvasive, potentially efficacious treatment for patients with notalgia paresthetica with cryolipolysis is warranted. Single or sequential cryolipolysis treatments may be required. The extent and duration of anticipated clinical resolution of notalgia paresthetica symptoms remain to be observed. Depending on the individual’s notalgia paresthetica-related cutaneous symptoms, complete (or partial) resolution of their notalgia paresthetica could be expected following cryolipolysis.
